# Geography, inequities, and the social determinants of health in transplantation

**DOI:** 10.3389/fpubh.2023.1286810

**Published:** 2023-12-07

**Authors:** Katherine Ross-Driscoll, Lisa M. McElroy, Joel T. Adler

**Affiliations:** ^1^Department of Surgery, Indiana University School of Medicine, Indianapolis, IN, United States; ^2^Center for Health Services Research, Regenstrief Institute, Indianapolis, IN, United States; ^3^Department of Surgery, Duke University School of Medicine, Durham, NC, United States; ^4^Division of Abdominal Transplant Surgery, Department of Surgery and Perioperative Care, Dell Medical at the University of Texas at Austin, Austin, TX, United States

**Keywords:** geography, inequities, SDOH, social determinants of health, transplantation

## Abstract

Among the causes of inequity in organ transplantation, geography is oft-cited but rarely defined with precision. Traditionally, geographic inequity has been characterized by variation in distance to transplant centers, availability of deceased organ donors, or the consequences of allocation systems that are inherently geographically based. Recent research has begun to explore the use of measures at various geographic levels to better understand how characteristics of a patient’s geographic surroundings contribute to a broad range of transplant inequities. Within, we first explore the relationship between geography, inequities, and the social determinants of health. Next, we review methodologic considerations essential to geographic health research, and critically appraise how these techniques have been applied. Finally, we propose how to use geography to improve access to and outcomes of transplantation.

## 1 Introduction

Geography plays an important role in shaping social, economic, governmental, and cultural influences of history, helping us to understand both space and place. Much attention in solid organ transplantation has focused on how to use geography to connect diverse fields of knowledge, such as epidemiology, medicine, biostatistics, and spatial analysis, to improve the clinical practice of transplantation (1). Recently, geographic research in the field has focused on measuring, defining, and explaining how inequities arise in availability and allocation of deceased donor organs, access to the transplant waitlist, and post-transplant outcomes.

Space and place are necessarily intertwined in the delivery of highly specialized care such as solid organ transplantation (2). The physical infrastructure, distribution, and specialization of resources play a significant role in transplantation: transplant centers tend to be located in urban settings inside tertiary and quaternary care centers, and allocation policy is shaped by transportation considerations weighed against cold ischemia time. Meanwhile, organ availability, wait time for transplant, and transplant center practices all vary widely.

Geography is also a major driver of the distribution of the social determinants of health (SDOH), or the conditions in the environments in which people are born, live, learn, work, and play (3, 4). The SDOH are widely acknowledged to impact health outcomes, and inequitable distribution of material resources and exposure to health-compromising conditions in turn drive health inequity (5, 6). Studying spatial patterns of access to and outcomes of solid organ transplantation can provide important insights into both associative and causal mechanisms of health outcomes and health inequities in this field.

Tobler’s first law of geography notes, that “everything is related to everything else, but near things are more related than distant things” (7). Considering this, we will review selected methodological concepts in geographic health research and describe how these concepts impact the study of transplantation. Next, we critically appraise how research into geographic inequities has been utilized in the field of transplantation to date. Finally, we propose how to use geography in studies intended to improve access to and outcomes of transplantation.

## 2 Methodological considerations

### 2.1 The ecological fallacy

The “ecological fallacy” was most famously noted by Robinson in 1950, which showed that differing results could be obtained when the same dataset was analyzed at the individual and the aggregate level (8). The actual term “ecological fallacy” was coined in 1958, and cautionary tales of the ecological fallacy abound in the social and medical sciences (9). Advances in statistical approaches have given us multilevel modeling methods to explicitly consider cross-level processes, such as a neighborhood’s impact on individual health outcomes, without committing the ecological fallacy (10). These multilevel models can be applied to classic regression techniques, such as logistic regression, and allow for consideration residual components at each level in the hierarchy/levels – helping to avoid the ecological fallacy and come closer to a causal interpretation.

Despite these advances, there remains a deep skepticism of the use of area-level measures in clinical research when used to reflect conditions shared by an administrative or geographic boundary such as state, region, ZIP code, census tract, or block group. In fact, many papers that model individual-level health outcomes using multi-level regression still describe the ecological fallacy in their limitations sections (11, 12). The ecological fallacy now seems to be shorthand for concern around the validity and utility of area-level measures.

One source of this concern may be due to the interchangeable use of area-level measures to refer to both derived and integral measures (13). Derived measures are those that represent some aggregate value of the individuals residing within an area. A very common example of derived measures in health research is census-based measures of the socioeconomic characteristics of residents of a neighborhood, such as median household income, percent living in poverty, or percent living in overcrowded housing. In contrast, integral measures are those that are intrinsic to the physical or social environment of the neighborhood. Common examples include measures of green space in a neighborhood, the availability of health services (examples in transplantation include dialysis facilities, specific medical specialties such as gastroenterologists or nephrologists, and transplant centers), or measures of neighborhood safety and social cohesion. While derived measures tend to have an obvious parallel to an individual-level measure, integral measures do not.

Derived measures are more common in research than integral measures. Perhaps as a result, a common way to conceptualize area-level measures is as a proxy for individual-level experiences – for example, area-level socioeconomic status (SES) as a proxy for individual-level wealth (14). While area-level and individual-level socioeconomic status are undoubtedly linked, the substitution of one for the other implies that if individual-level SES were only collected, area-level SES could be dispensed with altogether. This simplification ignores the myriad impacts that area-level exposures can have on individual-level health outcomes and belies the lack of causal thinking that underpins much of the neighborhood effects research (often with area-level measures such as median household income, percent living under the poverty line, or rurality, as well as indices such as the Area Deprivation Index or the Social Vulnerability Index) in the field of transplantation.

To improve the rigor of this area of research, future work needs to incorporate principles of causal thinking as they relate to area-level determinants of health. Specifically, researchers need to grapple with identifying and understanding specific pathways through which area-level measures have an impact on an individual’s health. This may mean moving away from the growing reliance on “indices” of socioeconomic deprivation (15, 16) and focusing on integral measures that may mediate associations between these indices and health outcomes.

### 2.2 Issues of scale and the modifiable areal unit problem

In geographic sciences, scale refers to the spatial dimensions at which entities, patterns, and processes can be observed and characterized. Geographic scale specifically refers to the extent of an area of interest, or the amount of area over which a pattern can vary, while observation (or measurement) scale refers to the number and nature of the spatial units over which a pattern can be expressed (17). In spatial analysis, the size of areal unit directly determines the amount of detail to be included in the analysis and the results generated. Too small a scale will result in not enough data to be aggregated in each unit of analysis, while an overly large scale will lead to overgeneralization of the data and a loss of detail.

The importance of establishing a scale that balances level of detail and the degree of aggregation in geographical analysis is underscored by the modifiable areal unit problem (MAUP, Figure) (18). MAUP refers to the sensitivity of data to the spatial scale and is a source of statistical bias that can significantly impact results. MAUP has two forms: (1) the scale effect, which refers to the fact that using bigger or smaller analysis units will inevitably lead to different analysis results, and (2) the zoning problem, which refers to the differences caused by the division of the study area (e.g., polygons vs. circles) even at the same spatial scale (19).

MAUP is an inherent phenomenon in geographic research due to the arbitrary nature of boundaries applied to group populations. MAUP is highly applicable in transplant research due to the variety of study areas used for data analysis. These include individuals, census tracts, counties, states or nations, but also donor service area (DSA), organ procurement organization (OPO), or United Network for Organ Sharing (UNOS) region – or the analogous units in other countries throughout the world. Major policy initiatives that change geographic boundaries with respect to organ allocation increase the significance and challenge of MAUP, particularly for researchers conducting retrospective cohort studies that include points of geographic reorganization (20).

While largely inevitable, researchers can quantify and mitigate effects of MAUP. The most critical step is pre-analysis selection of the optimal scale for data aggregation. Explicit rationale for areal units used is essential for analytic planning and placing results in context. Several advanced spatial analytic techniques can aid in reducing the uncertainty introduced by MAUP. These include merging highly correlated spatial units, small area estimation, bootstrapping and geographically weighted regression (21, 22).

## 3 Geographic considerations specific to health equity research

The extent of aggregation and geographic scale should be carefully considered when planning a geographic analysis for the purposes of understanding and mitigating health inequities. Measures should be considered and aggregated based on level of influence appropriate for the research hypothesis being tested; a summary of some available measures are in the Table 1.

**Table 1:** Selected examples of area-level measures and associated inequities.

Unit	Area-level measure	Example inequities	References
Census tract	Area deprivation index	Survival after Covid-19	(23)
Variable, depending on country	European Deprivation Index	Increased risk of death after kidney transplantation	(16)
Zip code	Social deprivation	Waitlisting and transplant rates for pediatric liver patients	(24)
County	Rurality	Waitlisting for kidney, liver, heart transplant	(25)
		Kidney transplant rates	(26)
Donor Service Area	Market competition/Herfindahl Hirschman Index	Use of marginal kidneys, survival after transplantation	(27)
State	Medicaid expansion	Waitlist rates for racial and ethnic minorities	(28)

For international studies, national boundaries and population demographics may influence longitudinal analyses. Examining access to transplant at the national level may incur a large bias due to the heterogeneity of regions within national borders. Rurality, high traffic roads, broadband access are all reported nationally—and can be compared internationally—but vary dramatically within national boundaries. Specific to transplant, variation in organ allocation and transportation systems, population demographics, national prevalence of end stage organ disease, and payment models for solid organ transplant will introduce significant heterogeneity in assessments of equity in access to care (29, 30). All of these contribute to the possibility of ecological fallacy in transplantation research.

Within the United States, administrative boundaries such as UNOS regions or DSAs are often used as units of comparison. This is appropriate for studies of geographic inequities in access to organs or center behavior (27), but may be less appropriate when investigating access to the waitlist, waiting times or postoperative outcomes. Longitudinal retrospective studies should take care in decisions to include time periods before and after a major geographic redistricting when using administrative boundaries. OPOs do not correlate with any of the above levels, so must be assessed at the donor or center level. As allocation in the United States has changed to circles around the donor hospital, the metrics of access to donors have reverted to nautical miles (31, 32).

State level aggregation may be particularly relevant for research investigating payment models, large employer-based payors, and Medicaid coverage which sometimes restricts payment to in-state. State-level legislation provides payment for transplant for undocumented immigrants and living donor benefits (33, 34). Some states also control transportation and broadband access and provide additional social relief programs not specific to transplant. County level aggregation is seen less commonly in transplant-related research, but many social safety net programs are county funded. Common measures aggregated at the county level are life-expectancy rate and infant mortality rate.

Center-level aggregation is in some ways the most salient to health equity research in transplant when examining referral and listing patterns. Recent work in the United States has suggested value in the transplant referral regions (TRR) (35–37)—based on patient migration patterns towards specific transplant centers. In some areas, a TRR corresponds to an entire state (Nebraska, Mississippi, and Alabama, for example); however, the TRRs are irregular and it is difficult to extrapolate findings from single-center, single-state regions to multi-center or multi-state regions. Other boundaries available include metropolitan service areas and hospital service areas. All of these measures need to consider, in some way, the degree of rurality in each of the units.

For transplant patients, geographic area of residence may be used to assess social risk via community resources/deprivation through indices, public databases, or demographic composition of neighborhoods. Here ZIP code and address (census tract or block group) are often used units of aggregation. ZIP codes are also administrative units established for mail delivery rather that to characterize populations, and their use to characterize neighborhood environment introduces bias with regards to the population size and association with greater heterogeneity than census tracts or block groups, particularly in areas of high population density. ZIP codes may cut across census block groups and lead to misclassification of structural social determinants of health and over or underestimation of community deprivation (38–40). Aggregation at the census tract or block group may also lead to over or underestimation of social risk of an individual/family/network or confounding by indication where minoritized neighborhoods may be associated with uniformly low SES status: an example is the Hyde Park neighborhood of Chicago, which has primarily black residents, some very impoverished, and some very wealthy.

## 4 Controversies and future directions

The multilevel nature of health inequities impels researchers to include measures that reflect not just individual, but community, center and national characteristics. Geographic analyses are not new, but have become more sophisticated in their methods and gained significant momentum in solid organ transplant. Appropriate use of geographic measures can help elucidate how interactions between patients, transplant centers, and the communities they reside in influence equity in access to and outcomes after transplant. Critical aspects of these studies include incorporation of geographic units into hypothesis generation, careful attention to the unit of aggregation of primary and secondary data, and purposeful use of composite measures of individual social determinants of health.

In the transplant literature, various social deprivation indices have been employed in an attempt to understand how neighborhood disadvantage mediates waitlist and transplant outcomes (41). There are multiple well-known deprivation indices but no universally accepted metric to capture social-contextual risk factors across geographical areas. Studies of neighborhood deprivation and its association with inequities in access to transplant have been inconsistent in choice of deprivation index, with little explanation of the relation to the specific index to the study hypothesis. As described above, the use of neighborhood-level deprivation as a proxy for patient-level socioeconomic status is problematic because of the ecological fallacy. These can measure two different, albeit related, aspects of the causal mechanism and should be described separately when possible.

However, these indices can also describe neighborhood access to material resources and exposure to health-deteriorating environmental factors, which can have an impact on health outcomes above and beyond individual-level socioeconomic status. Greater attention is needed to the intermediate causal mechanisms behind the associations between neighborhood deprivation indices and transplant health outcomes in order to inform clinical and policy interventions.

Ultimately, using geography as a method to understand inequities, both at the individual and area level, is a useful and powerful method to improve access to transplantation. Its use requires careful consideration and proper planning to avoid unrecognized bias and resultant inaccurate conclusions. Space, place and health are undeniably intertwined. Geography will continue to play a prominent role in driving health equities. As methods of geographic analysis advance, so should our understanding of how the macro and microenvironment influence access to and outcomes after solid organ transplant.

## Author contributions

KR-D: Writing – original draft, Writing – review & editing. LM: Writing – original draft, Writing – review & editing. JA: Conceptualization, Writing – original draft, Writing – review & editing.

## References

[1] Salvalaggio PR. Geographic disparities in transplantation. Curr Opin Organ Transplant. (2021) 26:547–53. doi: 10.1097/MOT.0000000000000914 34411039

[2] Ross-Driscoll K Patzer RE Axelrod DA. Ecological factors and posttransplant outcomes: causation or correlation? Am J Transplant. (2021) 21:3219–20. doi: 10.1111/ajt.16716, PMID: 34117719 PMC9051979

[3] Marmot M Allen JJ. Social determinants of health equity. Am J Public Health. (2014) 104:S517–9. doi: 10.2105/AJPH.2014.302200, PMID: 25100411 PMC4151898

[4] Butler SM. What is the outlook for addressing social determinants of health? JAMA Health Forum. (2021) 2:e213639. doi: 10.1001/jamahealthforum.2021.3639 36218664

[5] Chan NW Moya-Mendez M Henson JB Zaribafzadeh H Sendak MP Bhavsar NA. Social determinants of health data in solid organ transplantation: national data sources and future directions. Am J Transplant. (2022) 22:17096. doi: 10.1111/ajt.17096 PMC9547872 35583111

[6] Park C Jones MM Kaplan S Koller FL Wilder JM Boulware LE. A scoping review of inequities in access to organ transplant in the United States. Int J Equity Health. (2022) 21:22. doi: 10.1186/s12939-021-01616-x 35151327 PMC8841123

[7] Tobler W. A computer movie simulating urban growth in the Detroit region. Econ Geogr. (1970) 46:234–40. doi: 10.2307/143141

[8] Robinson WS. Ecological correlations and the behavior of individuals. Am Sociol Rev. (1950) 15:351–7. doi: 10.2307/2087176 19179346

[9] Subramanian SV Jones K Kaddour A Krieger N. Revisiting Robinson: the perils of individualistic and ecologic fallacy. Int J Epidemiol. (2009) 38:342–60. doi: 10.1093/ije/dyn359, PMID: 19179348 PMC2663721

[10] Merlo J. A brief conceptual tutorial of multilevel analysis in social epidemiology: using measures of clustering in multilevel logistic regression to investigate contextual phenomena. J Epidemiol Community Health. (2006) 60:290–7. doi: 10.1136/jech.2004.029454 16537344 PMC2566165

[11] Volk ML Choi H Warren GJW Sonnenday CJ Marrero JA Heisler M. Geographic variation in organ availability is responsible for disparities in liver transplantation between Hispanics and Caucasians. Am J Transplant. (2009) 9:2113–8. doi: 10.1111/j.1600-6143.2009.02744.x, PMID: 19624565

[12] Snow KK Patzer RE Patel SA Harding JL. County-level characteristics associated with variation in ESKD mortality in the United States, 2010-2018. Kidney360. (2022) 3:891–9. doi: 10.34067/KID.0007872021 36128479 PMC9438422

[13] Oakes JM. The (mis) estimation of neighborhood effects: causal inference for a practicable social epidemiology. Soc Sci Med. (2004) 58:1929–52. doi: 10.1016/j.socscimed.2003.08.004 15020009

[14] Diez Roux AV. The study of group-level factors in epidemiology: rethinking variables, study designs, and analytical approaches. Epidemiol Rev. (2004) 26:104–11. doi: 10.1093/epirev/mxh006 15234951

[15] Kind AJH Buckingham WR. Making neighborhood-disadvantage metrics accessible — the Neighborhood atlas. N Engl J Med. (2018) 378:2456–8. doi: 10.1056/NEJMp1802313, PMID: 29949490 PMC6051533

[16] Châtelet V Bayat-Makoei S Vigneau C Launoy G Lobbedez T. Renal transplantation outcome and social deprivation in the French healthcare system: a cohort study using the European deprivation index. Transpl Int. (2018) 31:1089–98. doi: 10.1111/tri.13161, PMID: 29611277

[17] Oshan TM Wolf LJ Sachdeva M Bardin S Fotheringham AS. A scoping review on the multiplicity of scale in spatial analysis. J Geogr Syst. (2022) 24:293–324. doi: 10.1007/s10109-022-00384-8

[18] Chen L Gao Y Zhu D Yuan Y Liu Y. Quantifying the scale effect in geospatial big data using semi-variograms. PLoS One. (2019) 14:e0225139. doi: 10.1371/journal.pone.0225139 31725781 PMC6855636

[19] Clark CR Williams DR. Understanding County-level, cause-specific mortality: the great value-and limitations-of small area data. JAMA. (2016) 316:2363–5. doi: 10.1001/jama.2016.12818, PMID: 27959983

[20] Hardeman RR Homan PA Chantarat T Davis BA Brown TH. Improving the measurement of structural racism to achieve antiracist health policy. Health Aff. (2022) 41:179–86. doi: 10.1377/hlthaff.2021.01489 PMC9680533 35130062

[21] Tuson M Yap M Kok MR Murray K Turlach B Whyatt D. Incorporating geography into a new generalized theoretical and statistical framework addressing the modifiable areal unit problem. Int J Health Geogr. (2019) 18:6. doi: 10.1186/s12942-019-0170-3, PMID: 30917821 PMC6437958

[22] Haynes D Hughes KD Rau A Joseph AM. The effect of pre-aggregation scale on spatially adaptive filters. Spat Spatiotemporal Epidemiol. (2022) 40:100476. doi: 10.1016/j.sste.2021.100476 35120678 PMC10688538

[23] Swan JT Rizk E Jones SL Nwana N Nicolas JC Tran AT. Hospitalization and survival of solid organ transplant recipients with coronavirus disease 2019: a propensity matched cohort study. PLoS One. (2022) 17:e0278781. doi: 10.1371/journal.pone.0278781, PMID: 36534667 PMC9762563

[24] Wadhwani SI Ge J Gottlieb L Lyles C Beck AF Bucuvalas J. Racial/ethnic disparities in wait-list outcomes are only partly explained by socioeconomic deprivation among children awaiting liver transplantation. Hepatology. (2022) 75:115–24. doi: 10.1002/hep.32106, PMID: 34387881 PMC8934136

[25] Axelrod DA Guidinger MK Finlayson S Schaubel DE Goodman DC Chobanian M. Rates of solid-organ wait-listing, transplantation, and survival among residents of rural and urban areas. JAMA. (2008) 299:202–7. doi: 10.1001/jama.2007.50, PMID: 18182602

[26] Tonelli M Klarenbach S Rose C Wiebe N Gill J. Access to kidney transplantation among remote-and rural-dwelling patients with kidney failure in the United States. JAMA. (2009) 301:1681–90. doi: 10.1001/jama.2009.545, PMID: 19383959

[27] Adler JT Sethi RKV Yeh H Markmann JF Nguyen LL. Market competition influences renal transplantation risk and outcomes. Ann Surg. (2014) 260:550–7. doi: 10.1097/SLA.0000000000000896, PMID: 25115431

[28] Nephew LD Mosesso K Desai A Ghabril M Orman ES Patidar KR. Association of State Medicaid Expansion with Racial/ethnic disparities in liver transplant wait-listing in the United States. JAMA Netw Open. (2020) 3:e2019869. doi: 10.1001/jamanetworkopen.2020.19869, PMID: 33030554 PMC7545310

[29] Bayer F Dorent R Cantrelle C Legeai C Kerbaul F Jacquelinet C. France’s new lung transplant allocation system: combining equity with proximity by optimizing geographic boundaries through the supply/demand ratio. Transpl Int. (2022) 35:10049. doi: 10.3389/ti.2022.10049, PMID: 35686227 PMC9171509

[30] Audry B Savoye E Pastural M Bayer F Legeai C Macher MA. The new French kidney allocation system for donations after brain death: rationale, implementation, and evaluation. Am J Transplant. (2022) 22:2855–68. doi: 10.1111/ajt.17180 36000787

[31] Adler JT Husain SA King KL Mohan S. Greater complexity and monitoring of the new kidney allocation system: implications and unintended consequences of concentric circle kidney allocation on network complexity. Am J Transplant. (2021) 21:2007–13. doi: 10.1111/ajt.16441, PMID: 33314637

[32] Cron DC Husain SA Adler JT. The new distance-based kidney allocation system: implications for patients, transplant Centers, and organ procurement organizations. Curr Transplant Rep. (2022) 9:302–7. doi: 10.1007/s40472-022-00384-z 36254174 PMC9558035

[33] Ansell D Pallok K Guzman MD Flores M Oberholzer J. Illinois law opens door to kidney transplants for undocumented immigrants. Health Aff. (2015) 34:781–7. doi: 10.1377/hlthaff.2014.1192 25941279

[34] State Laws Impacting Living Organ Donation. (n.d.) National Kidney Foundation. Available at: https://voices.kidney.org/state-laws-impacting-living-organ-donation/ (Accessed January 20, 2023).

[35] Ross-Driscoll K Axelrod D Lynch R Patzer RE. Using geographic catchment areas to measure population-based access to kidney transplant in the United States. Transplantation. (2020) 104:e342–50. doi: 10.1097/TP.0000000000003369 33215901

[36] Ross-Driscoll K Gunasti J Lynch RJ Massie A Segev DL Snyder J. Listing at non-local transplant centers is associated with increased access to deceased donor kidney transplantation. Am J Transplant. (2022) 22:1813–22. doi: 10.1111/ajt.17044, PMID: 35338697 PMC9580509

[37] Schappe T Peskoe S Bhavsar N Boulware LE Pendergast J McElroy LM. Geospatial analysis of organ transplant referral regions. JAMA Netw Open. (2022) 5:e2231863. doi: 10.1001/jamanetworkopen.2022.31863, PMID: 36107423 PMC9478781

[38] Krieger N Waterman P Chen JT Soobader MJ Subramanian SV Carson R. Zip code caveat: bias due to spatiotemporal mismatches between zip codes and US census-defined geographic areas--the public health disparities geocoding project. Am J Public Health. (2002) 92:1100–2. doi: 10.2105/ajph.92.7.1100, PMID: 12084688 PMC1447194

[39] Cooper RA Cooper MA McGinley EL Fan X Rosenthal JT. Poverty, wealth, and health care utilization: a geographic assessment. J Urban Health. (2012) 89:828–47. doi: 10.1007/s11524-012-9689-3, PMID: 22566148 PMC3462827

[40] Sadler RC. Misalignment between ZIP codes and municipal boundaries: a problem for public health. Cityscape. (2019) 21:335–40. 34306299 PMC8301226

[41] Park C Schappe T Peskoe S Mohottige D Chan NW Bhavsar NA. A comparison of deprivation indices and application to transplant populations. Am J Transplant. (2023) 23:377–86. doi: 10.1016/j.ajt.2022.11.018, PMID: 36695687 PMC10226151

